# Identification and functional analysis of novel protein-encoding sequences related to stress-resistance

**DOI:** 10.3389/fmicb.2023.1268315

**Published:** 2023-09-28

**Authors:** Joshelin Huanca-Juarez, Edson Alexandre Nascimento-Silva, Ninna Hirata Silva, Rafael Silva-Rocha, María-Eugenia Guazzaroni

**Affiliations:** ^1^Department of Cell and Molecular Biology, Ribeirão Preto School of Medicine (FMRP) – University of São Paulo (USP), São Paulo, Brazil; ^2^Department of Biology, Faculty of Philosophy, Sciences and Letters of Ribeirão Preto (FFCLRP) – University of São Paulo (USP), São Paulo, Brazil; ^3^ByMyCell Inova Simples, São Paulo, Brazil

**Keywords:** stress resistance, bacterial robustness, industrial biotechnology, synthetic biology, metagenomics

## Abstract

Currently, industrial bioproducts are less competitive than chemically produced goods due to the shortcomings of conventional microbial hosts. Thus, is essential developing robust bacteria for improved cell tolerance to process-specific parameters. In this context, metagenomic approaches from extreme environments can provide useful biological parts to improve bacterial robustness. Here, in order to build genetic constructs that increase bacterial resistance to diverse stress conditions, we recovered novel protein-encoding sequences related to stress-resistance from metagenomic databases using an *in silico* approach based on Hidden-Markov-Model profiles. For this purpose, we used metagenomic shotgun sequencing data from microbial communities of extreme environments to identify genes encoding chaperones and other proteins that confer resistance to stress conditions. We identified and characterized 10 novel protein-encoding sequences related to the DNA-binding protein HU, the ATP-dependent protease ClpP, and the chaperone protein DnaJ. By expressing these genes in *Escherichia coli* under several stress conditions (including high temperature, acidity, oxidative and osmotic stress, and UV radiation), we identified five genes conferring resistance to at least two stress conditions when expressed in *E. coli*. Moreover, one of the identified HU coding-genes which was retrieved from an acidic soil metagenome increased *E. coli* tolerance to four different stress conditions, implying its suitability for the construction of a synthetic circuit directed to expand broad bacterial resistance.

## 1. Introduction

Industrial biotechnology focuses on working with biological systems, such as cell factories or enzymes, rather than using fossil raw materials to produce antibiotics, chemicals, and energy sources (Festel, 2020). As a result, it has attracted much attention for its environmentally friendly and sustainable methods, which could pave the way for a paradigm shift from petroleum to bio-based production (Yu et al., 2019). Therefore, this industry is positioned as a solution to critical global concerns such as climate change mitigation, food security, ocean pollution reduction, and biodiversity conservation (Rosemann and Molyneux-Hodgson, 2020). Microbial Cell Factories, however, are subjected to harsh extracellular conditions such as high temperature, low pH, and metabolite toxicity, resulting in lower productivity than generated on a laboratory scale and, consequently, higher production costs (Gong et al., 2017; Jiang et al., 2020). Highly robust and tolerable strains towards non-natural or even toxic substrates or products and abiotic conditions are required since they can utilize cheaper carbon and energy sources, such as waste residues or biomass hydrolysates (Blombach et al., 2022).

Abiotic stress conditions—such as those present in industrial bioprocesses—produce considerable cellular damage in non-adapted microorganisms, affecting the structure and stability of various biomolecules, including nucleic acids, membrane lipids, and proteins (Rothschild and Mancinelli, 2001; Merino et al., 2019). To counteract these damage conditions, cells activate different repair mechanisms, such as DNA/RNA repair proteins, proteases to remove unfolded proteins, and multifunctional enzymes, such as RecA, Hup, RecR, RecO, PriA, and RecN. These enzymes are involved in DNA condensation, removal of broken DNA, and DNA recombination repair (Grosjean and Oshima, 2007; Wang et al., 2011; Somayaji et al., 2022). There is also a subset of activated post-translationally protein under specific stress conditions known as molecular chaperones, which act in several processes involving cellular protein folding, unfolding, and homeostasis (Saibil, 2013; Voth and Jakob, 2017). Considering that protein denaturation is a persistent direct or indirect result of stress, chaperones are vital in defensive cellular mechanisms (Jacob et al., 2017).

In addition to the enzymes mentioned above, there are other proteins of particular relevance as protective biological agents against stress damage to essential macromolecules. For instance, the DNA-binding protein HU (histone-like protein) has been shown to bind with high specificity to aberrant DNA formations such as nicks and strand breaks to preserve its integrity (Kamashev and Rouviere-Yaniv, 2000). The Clp protease complex consists of a proteolytic component, ClpP, in association with any of the ATPases, ClpA or ClpX, granting substrate specificity (Wang et al., 1997; Khan et al., 2022). Proteases, in general, play an essential role in protein quality control by removing short-lived regulatory proteins as well as misfolded and damaged proteins, which are toxic to the cell (Yu and Houry, 2007). Lastly, DnaJ is a homolog of eukaryotic HSP40 and acts as a cofactor of DnaK, stimulating its ATPase activity through the J domain, while additional domains give specificity to the system (Cheetham and Caplan, 1998; Ghafoori et al., 2017; Kampinga et al., 2019). In addition to participating in other metabolic tasks, these molecular chaperones have been shown to play an essential role in protein degradation (Huang et al., 2001).

In recent years, various studies have focused on the capacity of bacteria to survive in extreme environments in order to understand the mechanisms of biochemical adaptation to harsh conditions (Morozkina et al., 2010; Guazzaroni et al., 2015; Mirete et al., 2016). Extremophiles live in a wide range of hostile conditions and have evolved a set of adaptations to cope with these situations (Coker, 2019). However, most of them are not cultivable, which limits their exploration in biotechnological applications (Rappé and Giovannoni, 2003; Alves et al., 2018b). Culture-independent approaches help harness the biological activity and build components of this difficult-to-grow biodiversity (van der Helm et al., 2018). In this context, functional and sequence-based metagenomics have been shown to be effective in the identification of novel genes that confer resistance to extreme conditions, such as enzymes, antibiotics, and other bioactive molecules derived from a variety of environments (Alves et al., 2018a). Functional metagenomic approaches allowed to identify novel genes relevant to biotechnology, involving the creation of expression libraries with thousands of metagenomic clones and activity-based screens in order to find genes that encode a function of interest (Guazzaroni et al., 2013; Mirete et al., 2016). On the other hand, the search for sequences of interest can also be done by sequence identification in (meta)genomic databases; however, low-level expression of heterologous proteins in commonly used hosts and low quality of (meta)genomic annotations are the main disadvantages in both approaches, respectively (Alves et al., 2018b; van der Helm et al., 2018). Furthermore, sequence-based screenings generally employ sequence pair aligners (such as BLAST, BLAT, Minimap2, KMA, and Bowtie) to detect regions of similarity between two biological sequences (Altschul et al., 1990; Kent, 2002; Langmead and Salzberg, 2012; Clausen et al., 2018). These tools constrain the homologous detection process since the percentage of identity is superior to a certain threshold, so more distant evolutionary sequences are lost (Skewes-Cox et al., 2014).

On the other hand, computational methods based on profiles, such as Hidden Markov Model (HMM), allow the detection of remote protein homologs (Kirsip and Abroi, 2019; Radivojac, 2022). In this context, profiling methods gained sensitivity by incorporating position-specific information into the alignment process and quantifying variation between family members at each position (Madera and Gough, 2002; Skewes-Cox et al., 2014). Some software based on HMM profiles were shown to be useful for identifying sequences of interest, such as IDOP or CryProcessor, used for detecting toxin genes (Shikov et al., 2020; Díaz-Valerio et al., 2021). In the same way, deep learning has been shown to be a powerful machine learning approach in predicting DNA sequence affinities and identifying new genes of interest, such as resistance genes or transcription factors (Arango-Argoty et al., 2018; Oliveira Monteiro et al., 2022). Considering the above, we aimed to identify microbial genes related to stress resistance by mining sequences from available metagenomic datasets from extreme environments using HMM profiles. For this achievement, we focused on searching novel protein sequences associated with the DNA-binding protein HU, the ATP-dependent protease ClpP, and the chaperone protein DnaJ. Then, we characterized their functional performance in *Escherichia coli* under five stress conditions (high temperature, acidity, oxidative and osmotic stress, and UV radiation). Through this *in silico* approach, we identified 10 new protein-encoding sequences related to stress-resistance that exhibited the potential to enhance resistance to various stress conditions in bacteria. These findings open up possibilities for the development of synthetic gene circuits aimed at broadening bacterial resistance capabilities.

## 2. Materials and methods

### 2.1. Bacterial strains and culture conditions

The bacterial strain *E. coli* DH10B was employed as a host in the stress resistance experiments. It was routinely grown in M9GlycAA (1x M9 salts, 2 mM MgSO_4_, 0.1 mM CaCl_2_, 0.1% casamino acids, 1% glycerol) under aerobic conditions at 37°C. Liquid cultures were cultivated in a shaker at 37°C and 200 rpm. For plasmid maintenance, the growth M9GlycAA was supplemented with 150 μg/mL ampicillin (Ap). For stress assays, overnight cultures were diluted to a final OD_600_ 0.05 in 5 mL of M9GlycAA with antibiotic and incubated in an orbital shaker at 37°C and 200 rpm. When cultures reached the early exponential growth phase (OD_600_ = 0.3–0.4), stress tests were initiated.

### 2.2. Selection of metagenomes from public databases

Raw shotgun metagenomes were curated, chosen, and downloaded from the MG-RAST database, hosted by the University of Chicago and the Argonne National Laboratory.^1^ The data were then pre-processed using FastQC to check the sequence’s quality, and when necessary, low-quality sequences were excluded using the Trimmomatic tool (Bolger et al., 2014). After that stage, metagenomes were assembled into contigs using the MEGAHIT program (Li et al., 2015), and an initial annotation was performed using Prokka.

### 2.3. Selection of genes of interest and construction of HMM profiles

HMM profiles were built based on selected stress-resistance genes previously documented in the literature. For this, we selected genes of nucleic acid binding protein genes (RBP, HU, DPS); DNA replication protein genes (GyrA, RecA, DnaA), chaperone and protease genes (ClpP, ClpX, ClpA, ClpC, ClpE, ClpL, DnaJ, DnaK); and CRISPR system genes (Cas1, Cas2, Cas9). The nucleotide sequences were obtained from the National Center for Biotechnology Information (NCBI) database, and alignment was performed with MAFFT (Multiple Alignment using Fast Fourier Transform) (Katoh et al., 2002). The profiles were created using the HMMer3 tool (Eddy, 2009), which was also used to detect homologous sequences by comparing the HMM profiles generated and the sequences of previously selected metagenomes. The results were analyzed and correlated with the literature, aiming to obtain 10 candidate genes. The selection process of protein sequences for further functional characterization was based on the following characteristics: (1) abundance of the proteins in the different environments, (2) metagenomic genes related to stress resistance in the literature, and (3) protein function. To continue the analysis and selection of sequences, a BLASTp was performed in order to identify the protein identity percentage.^2^

### 2.4. *In silico* analysis and 3D-structure model generation

Dendrograms for each class of protein were constructed based on the 23–25 amino acid sequences using MEGA X software. The homologous sequences of selected proteins in different organisms from the NCBI database were retrieved in FASTA format. Alignment was done using the Muscle algorithm (Edgar, 2004) and used to build the dendrogram. The Neighbor-joining statistical method was applied, and the accuracy of the phylogenetic analysis was predicted by using 1,000 bootstrapping replications. The 3D-structure models were generated using the Alphafold2 collab server (Jumper et al., 2021). All structures were analyzed using the PyMol v. 2.5.2 software^3^ where the RMSD value of the alpha carbon atoms was considered. Proteins were aligned with T-Coffee v. 13.45.60.cd84d2a, and the alignment results were visualized in Jalview v. 2.11.2.3.

### 2.5. Cloning of the selected genes

All amino acid sequences previously selected by bioinformatics analysis were converted to nucleotides sequences and optimized for expression in *E. coli* using the EMBOSS Backtranseq tool.^4^ Each nucleotide sequence was cloned under control from the constitutive promoter BBBa_J23106 (*Pj106*) and the ribosome binding site (RBS) BBa_B0034. Promoter and RBS sequences were obtained from the iGEM Foundation Standard Biological Parts Catalog (Registry).^5^ The genetic construct was assembled into the pUC19 vector. The synthesis of all plasmids containing the promoter, RBS, and candidate genes cloned into the pUC19 vector was requested from the company Integrated DNA Technologies (IDT). *E. coli* DH10B competent cells were transformed with the resulting plasmids.

### 2.6. Growth rate measurement and fitness cost calculation

Cell growth was monitored using a Victor X3 plate reader (PerkinElmer, Inc.), as described by de Siqueira et al. (2020). Microbial cultivation was evaluated at 30°C for 8 h. During this incubation period, the optical density at 600 nm was measured every 30 min. All experiments were performed with four biological replicates. For fitness cost calculation we followed the methodology described by de Siqueira et al. (2020).

### 2.7. Heat shock experiments

Exponential phase cultures of *E. coli* DH10B (OD_600_ = 0.3–0.4) carrying an empty plasmid or the pUC19 vector with *hup*, *clpP*, or *dnaJ* genes were diluted at 10^−1^ in PBS buffer (pH 7.2). Then, dilutions were incubated at 55°C, and aliquots were removed at 0 and 30 min. To determine CFU/mL, serial dilutions were made, and three 25 μL droplets (technical replicates) were placed onto M9GlycAA agar with Ap. Plates were incubated at 37°C for 24 h. Survival percentage was expressed as the number of CFU/mL remaining after heat treatment divided by the initial CFU/mL. *E. coli* DH10B carrying empty pUC19 plasmid was used as a negative control. Each experiment was performed at least three times.

### 2.8. Acid resistance assay

Exponential phase cultures of *E. coli* DH10B (OD600 = 0.3–0.4) carrying an empty plasmid or the pUC19 vector with *hup*, *clpP*, or *dnaJ* genes were diluted at 10^−1^ in PBS buffer (pH 7.2) or M9GlycAA acidified with HCl (pH = 3.0) and then incubated at 37°C. Aliquots were taken at 0 and 60 min, respectively. To determine CFU/mL, serial dilutions were plated and enumerated as previously described. Survival percentage was the number of CFU/mL remaining after acidity treatment divided by the CFU/mL at zero time. *E. coli* DH10B carrying empty pUC19 plasmid was used as a negative control. Each experiment was repeated at least three times.

### 2.9. Oxidative stress experiments

Exponential phase cultures of *E. coli* DH10B (OD600 = 0.3–0.4) carrying an empty plasmid or the pUC19 vector with *hup*, *clpP*, or *dnaJ* genes were diluted at 10^−1^ in PBS buffer (pH 7.2) or M9GlycAA + hydrogen peroxide 12 mM and incubated at 37°C. Aliquots were taken at 0 and 60 min, respectively. Survival percent was calculated as above. *E. coli* DH10B carrying empty pUC19 plasmid was used as a negative control. Each experiment was repeated at least three times.

### 2.10. UV radiation experiments

Overnight cultures of *E. coli* DH10B carrying an empty plasmid or a pUC19 vector with *hup*, *clpP*, or *dnaJ* genes were diluted in PBS buffer to 1.0 OD_600_. For UV radiation exposure, 10 μL of each culture were linearly spread onto M9GlycAA plates and irradiated with a germicidal lamp for 0, 5, 10, 15, 20, and 25 s at room temperature. Bacterial growth was evaluated after 24 h of incubation at 37°C. Cell growth up to a certain level of exposure was determined by counting at least 10 colonies in the spot. *E. coli* DH10B carrying empty pUC19 plasmid was used as a negative control. Each experiment was repeated at least three times.

### 2.11. Osmotic stress assay

For continuous NaCl exposure experiments, cells were cultivated in M9GlycAA medium +3.5% NaCl. Cultures were placed in a 96-well plate and incubated at 30°C for 8 h. During this incubation period, the optical density at 600 nm was measured every 30 min using a Victor X3 plate reader (PerkinElmer, Inc.). Growth rate was calculated as indicated above. Untransformed *E. coli* DH10B was used as a negative control. All experiments were performed with four biological replicates.

### 2.12. Data analysis and visualization

Data analysis and generation of growth curves were performed using the R statistical platform (version 4.2.0), using packages *ggpubr* and *ggplot2*. The *mipreadr* package version 0.1.0 was used to import and process the raw data produced by the plate reader. A Student’s *t*-test with a 5% significance level was employed to compare the values obtained. For clustering construction, we provide discrete arbitrary values to the different stress responses presented by clones for comparative purposes. Thus, data were classified as high, medium, and low-stress responses, and values of 2, 1, and 0 were given, respectively. Then, hierarchical clustering and normalization of data were performed using the R statistical platform (version 4.2.0).

## 3. Results and discussion

### 3.1. *In silico* screening of stress-related genes

**Figure 1:**
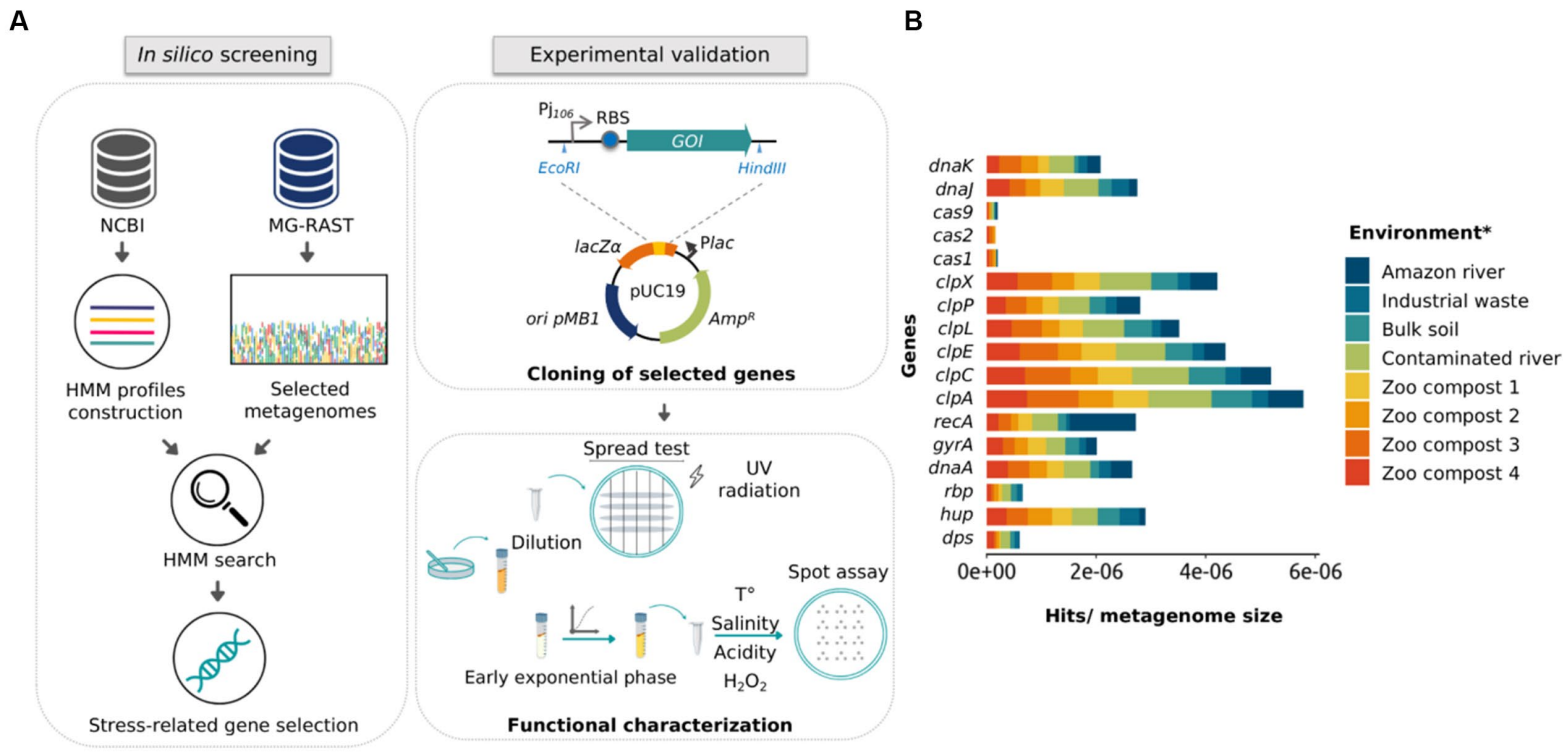
Schematic representation of the approach used in this study and the frequency of stress-related proteins in public metagenomic databases. **(A)** Schematic representation of *in silico* and experimental approaches used to identify and validate stress-related genes using HMM profiles, metagenomic mining, and experimental characterization. **(B)** Representation of gene frequency in different metagenomes selected from public databases. Frequency calculation was made by normalizing the number of found hits by the size of the metagenomic library (Gb). *Metagenomes from the Atacama Desert and Dourados are not presented in the **(B)** panel, since these metagenomic data did not include all classes of the proteins plotted.

The search for new stress-resistance genes is a useful way for expanding robustness in microorganisms of industrial interest (Chen and Jiang, 2018; Wehrs et al., 2019). Although metagenomics offers an excellent alternative for discovering novel functional genes, activity-driven screenings are time-consuming and present low recovery rates (Guazzaroni et al., 2015; van der Helm et al., 2018). On the other hand, sequence-driven screening depends on the quality of the sequences—which present high annotation errors—and does not allow the discovery of new sequences if they have not been previously related to a specific function (Alves et al., 2018b). In turn, profile search methods are more sensitive than pairwise alignment in detecting low percent identity homologs by incorporating position-specific information into the alignment process and quantifying variation across family members at each position (Madera and Gough, 2002; Skewes-Cox et al., 2014). Considering that, we addressed the identification of new genes in metagenomic databases using an *in silico* approach based on HMM profiles. The general computational pipeline began searching metagenomic shotgun sequencing data from 10 microbial communities of extreme environments recruited from the MG-RAST database. Then, we aimed to identify genes encoding chaperones and other proteins—such as proteases and nucleic acid-binding proteins—that confer resistance to stress conditions (Figure 1A).

As the first step for selecting the metagenomes, we selected those who met the following requirements: (i) that were sequenced through the Illumina platform; (ii) that the sequences did not belong to DNA from the microbiota of humans/animals; (iii) that the size of the studies was around 1 gigabyte of DNA data, and; (iv) that metagenomes were originated from environments presenting harsh conditions (or in which there was some marked abiotic stress). Selected metagenomes are listed in Supplementary Table S1, it should be noted that about 85% are from the Brazilian territory. A summary of the metagenome assembly metrics returned, and the annotation results are shown in Supplementary Tables S2, S3, respectively. Next, to create an HMM profile, a multiple sequence alignment of 17 different stress-related proteins deposited in the NCBI database was performed using the HMMer3 server (Supplementary Figure S1). Then, metagenome data were searched against those HMM probabilistic profiles. The resulting profiles were compared with annotated protein regions, generating a total of 6,840 hits (Supplementary Table S4).

For the selection of protein sequences, it was considered that a protein found in a broader range of environments would be linked to the response to more than one stress factor. Figure 1B shows the abundance of the 17 protein families in the assembled metagenomes, considering the number of hits normalized by the size of the corresponding metagenome. The graph shows that the *rbp* and *dps* genes had the lowest abundance, being disregarded in the subsequent analysis. Then, we carried out an extensive literature search and found that 50%–70% of the recovered published works connected *hup*, *clpP*, *clpX*, *dnaK*, and *dnaJ* genes to stress-defense mechanisms in microbial cells. As mentioned before, ClpP and ClpX protease subunits were reported to work together to produce a complex that degrades misfolded proteins and protects cells against its toxic effect (Baker and Sauer, 2012). However, different studies demonstrated that the ClpP protease subunit can function independently of the ATPase subunit ClpX (Guazzaroni et al., 2013; de Siqueira et al., 2020). On the other hand, the overexpression of the DnaK protein in *E. coli* presents a bactericidal effect (Blum et al., 1992; Jung and Ahn, 2022). Considering that, three families of proteins were selected for functional characterization, HU, ClpP, and DnaJ. To select the most promising protein sequences from the total sequences retrieved by the HHM approach, we evaluated if the chosen sequences were non-truncated when compared to homologous protein sequences using BLASTp. As a result, 24 protein sequences were selected (15 for HU, 6 for ClpP, and 3 for DnaJ), with protein identity percentage of 50%–85% for HU, 83%–97% for ClpP, and 55%–93% for DnaJ (Table 1).

**Table 1:** Summary of the main characteristics of the selected proteins.

Protein name	Source	Length (aa)	Identity percentage	Closest related microorganism^a^
HU.d1	Dourados	91	76	*Thermoleophilia bacterium*
HU.d2	Dourados	88	74	*Solirubrobacterales bacterium*
HU.jz	Juazeiro	90	60.23	*Sphingomonas koreensis*
HU.go	mgpGO	90	70.79	Unclassified bacterium
ClpP.da	Atacama Desert	198	97.92	*Rubrobacteraceae bacterium*
ClpP.jz	Juazeiro	193	83.87	*Candidatus Saccharibacteria bacterium*
ClpP.z4	Zoo 4	203	93.88	*Actinobacteria bacterium*
DnaJ.tt	Tietê	375	55.65	*Rhizobium* sp. *ARZ01*
DnaJ.z4	Zoo 4	389	88.11	Unclassified bacterium
DnaJ.jz	Juazeiro	379	93.90	*Thermomonas aquatica*

^a^Considering the closest similar protein according to BLASTp hits.

### 3.2. Sequence analysis of selected protein candidates

The 24 selected amino acid sequences were used to construct dendrograms for each protein family. This analysis allowed us to categorize the sequences according to protein sequence identity and select them based on their position resulting in the dendrogram (Supplementary Figures S2–S4). For this, we took into account as selection criteria (i) sequences presenting a lower percentage of amino acid identity; (ii) that belong to different environmental sources, and; (iii) that were located in different branches of the dendrogram. Those observations enabled us to choose 10 sequences for further functional characterization (Table 1). To verify the presence of conserved residues, sequence alignments (Supplementary Figure S5) and 3D structure models were constructed (Supplementary Figure S6). In the HU sequence alignment, the presence of proline 63 in HU sequences was verified (Supplementary Figure S5A), a highly conserved residue at the ends of the beta strands that surrounds the DNA and which, as part of an Arg-Asn-Pro motif (RNP), stabilizes the twists of the DNA (Grove, 2011). Interestingly, HU.d1 and HU.d2 have valine instead of arginine at position 61. The presence of Arg does not contribute to the stabilization of DNA twists, however, in *Thermotoga maritima*, this motif contains Val instead of Arg, therefore its removal appears to be required to maintain any distinction between perfect duplexes and damaged DNA (Grove, 2011). Ligation to perfect duplexes DNA would allow HU protein to protect them before exposure to stress, thus reducing damage. Additionally, the conserved glycine at position 15 was also observed, a residue that mediates loop flexibility and promotes the thermal stability of HU (Kawamura et al., 1996; Georgoulis et al., 2020) (Supplementary Figure S5A). Likewise, the presence of the Ser-His-Asp catalytic triad was observed in ClpP sequences, a motif fully conserved in members of the ClpP family and which plays an essential role in the cleavage capacity of proteases (Khan et al., 2022) (Supplementary Figure S5B). Similarly, the conserved His-Pro-Asp motif in DnaJ sequences was verified (Supplementary Figure S5C). This conserved motif recognizes and stimulates the ATPase activity of DnaK (Ahmad et al., 2011). We observed that the percentage of identity ranged from 60 to more than 90% in the 10 selected sequences when compared to proteins from BLASTp hits (Table 1), something that in principle would indicate that there is no novelty in the sequences. However, it should be noticed that none of these sequences have been experimentally characterized in previous studies.

Next, we carried out the cloning of the 10 selected sequences in individual genetic constructs (Supplementary Table S5). For this, each nucleotide sequence was cloned under control of the constitutive medium expression promoter Pj*_106_* and the RBS BBa_B0034, which was reported to present medium translation strength. Constructions were assembled into the pUC19 vector and resulting plasmids were transformed into *E. coli* DH10B (Figure 1A).

### 3.3. Metabolic burden evaluation of the genetic constructs

**Figure 2:**
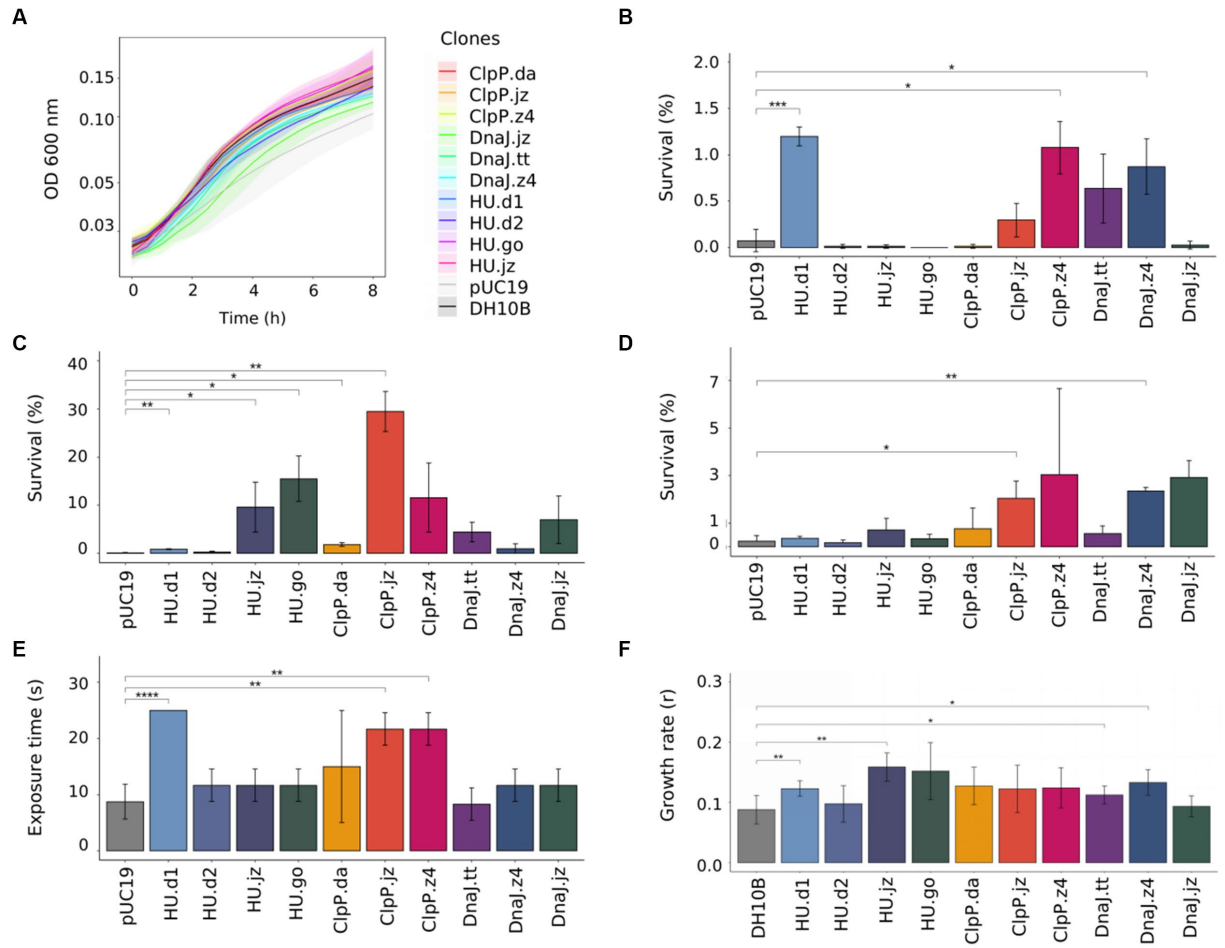
Functional characterization of *E. coli* DH10B clones carrying *hup*, *clpP,* and *dnaJ* genes. **(A)** Growth curve of *E. coli* DH10B clones in liquid M9GlycAA medium during 8 h; optical density at 600 nm was measured every 30 min (a 2.5× factor should be applied due to the optical path length). To test the effects of stress factors, *E. coli* DH10B clones were grown to exponential phase at 30°C, then **(B)** treated at 55°C for 30 min, **(C)** exposed to acid medium (pH = 3.0) for 60 min or **(D)** to 12 mM hydrogen peroxide for 60 min. Serial dilutions were made and CFU counts were used to determine the survival percentage. *E. coli* DH10B carrying empty pUC19 plasmid was used as a negative control. **(E)** Effect of UV radiation resistance in *E. coli* clones. Overnight cultures of *E. coli* clones were irradiated with a germicidal lamp (254 nm UV) during 0, 5, 10, 15, 20, and 25 s. **(F)** Effect of salinity on growth rates of *E. coli* DH10B clones. Growth rates were determined from the exponential curve fitting function as described in Section 2. *E. coli* DH10B was used as a negative control. Student’s *t*-test was used for comparison of the values obtained in the experiments and significance determination (*p* > 0.05; *p ≤ 0.05; **p ≤ 0.01; ***p ≤ 0.001; ****p ≤ 0.0001). All graphs represent the average from at least three independent experiments. Standard deviation from experiments is represented as shaded regions in **(A)** and vertical bars in **(B–F)**.

Considering that metabolic load, defined as the proportion of a host cell’s resources—energy molecules or carbon building blocks—that are used to express heterologous proteins, has an important impact in normal cellular processes (Glick, 1995; Wu et al., 2016), we proceeded to evaluate the metabolic burden of the 10 genetic constructs by measuring the growth of the clones in a culture medium without any stress factor. For this, *E. coli* DH10B clones bearing empty plasmid or genetic constructs were grown in M9GlycAA medium (supplemented with Ap when strains carried a plasmid), and the fitness cost was calculated considering the growth rates (Figure 2A; Supplementary Table S6). Unexpectedly, the clone carrying the empty pUC19 plasmid showed the highest fitness cost (42.1%). In accordance with this observation, there are several reports found in the literature or in research discussion forums that describe difficulties related to the growth or transformation efficiency of *E. coli* cells transformed with empty plasmid pUC19 (Toh, 2013; Amirie et al., 2014). This is probably due to a higher number of copies of this plasmid when it is not bearing extra genes, which imposes a high metabolic cost on the cell. To circumvent this limitation, we performed the experiments with clones (bearing empty plasmid or genetic constructs) situated in the same metabolic state, that is, stress tests were just initiated when cultures reached the early exponential growth phase (OD600 = 0.3–0.4). Also, for comparison purposes, untransformed *E. coli* cells were used as an additional negative control.

### 3.4. Response to stress conditions conferred by the stress-related genes

Microorganisms resistant to several stresses play an essential role in industrial bioprocesses because they permit increased yields and lower production costs (Thorwall et al., 2020; Wang et al., 2022). Production processes generally involve osmotic (e.g., processes involving high salt concentration culture medium), high temperature (such as processes that enable non-sterilized fermentation), acidity (e.g., organic acid production), and oxidative stresses (e.g., accumulation of radical oxygen species) (Patel et al., 2006; Qin et al., 2009; Li et al., 2011; Auesukaree, 2017; Şanlier et al., 2019; Ye and Chen, 2021). Similarly, bioremediation of polluted environments is also affected by different abiotic parameters since wastewater is produced by various industries such as chemical, petrochemical, and textile (Le Borgne et al., 2008; Wernick et al., 2016; Koshlaf and Ball, 2017; Aliko et al., 2022). Another critical application of stress-resistant microorganisms is in enzyme production processes, in which bacterial hosts need to be active and stable at high salt contents or high temperatures (Margesin and Schinner, 2001; Verma et al., 2021).

In order to study if genetic constructs—bearing independent new sequences coding for the DNA-binding protein HU, the ATP-dependent Clp protease proteolytic subunit, and the molecular chaperone DnaJ—increase *E. coli* resistance to diverse stress conditions, we performed stress shocks assays with *E. coli* clones in five stress conditions: high temperature, acidity, oxidative and osmotic stress, and UV radiation (Figure 2; Supplementary Table S7).

Considering the set of assays in the five stress conditions, we observed that expression of most of the encoding sequences granted stress resistance to the cells in differing degrees (Figure 2). For example, cells expressing *hu.d1* showed a positive response to four stress factors over the ones expressing either of the other hu homologs, with an average survival percentage of over 1.2% in heat shock tests (Figure 2B) and 0.8% in the acidic challenge (Figure 2C). These values represented a fold change of 17 and 9, respectively, in comparison to *E. coli* cells harboring the empty plasmid (0.07% in heat shock assays and 0.1% in acid shock experiments). These results agree with the previous work of Ogata et al. (1997), which showed that the HU protein maintains the negative supercoiling of DNA during thermal stress and contributes to cellular thermotolerance in *E. coli*. Similarly, the HU role in acid resistance was also corroborated by several studies (Bi et al., 2009; Guazzaroni et al., 2013; de Siqueira et al., 2020; Alves et al., 2023). In the UV radiation tests, cells expressing *hu.d1* also showed a higher percentage of survival (colony growth until 25 s of UV radiation exposure) compared with control cells (growth until 10 s) (Figure 2E; Supplementary Figure S7). This is probably because HU acts against UV radiation stress by preventing the formation of photoproducts and in the RecA-dependent repair process (Miyabe et al., 2000). At the same time, in the salinity assays, cells expressing *hu.d1* reached a growth rate of 0.12. This rate was 33% higher than that achieved by untransformed *E. coli* cells (Figure 2F; Supplementary Figure S8). The HU protein response to osmotic stress could be involve in the modulation of expression of sigma factor *rpoS* (σ38), a global regulator whose translation is stimulated in response to high osmolarity (Muffler et al., 1996; Battesti et al., 2011).

**Figure 3:**
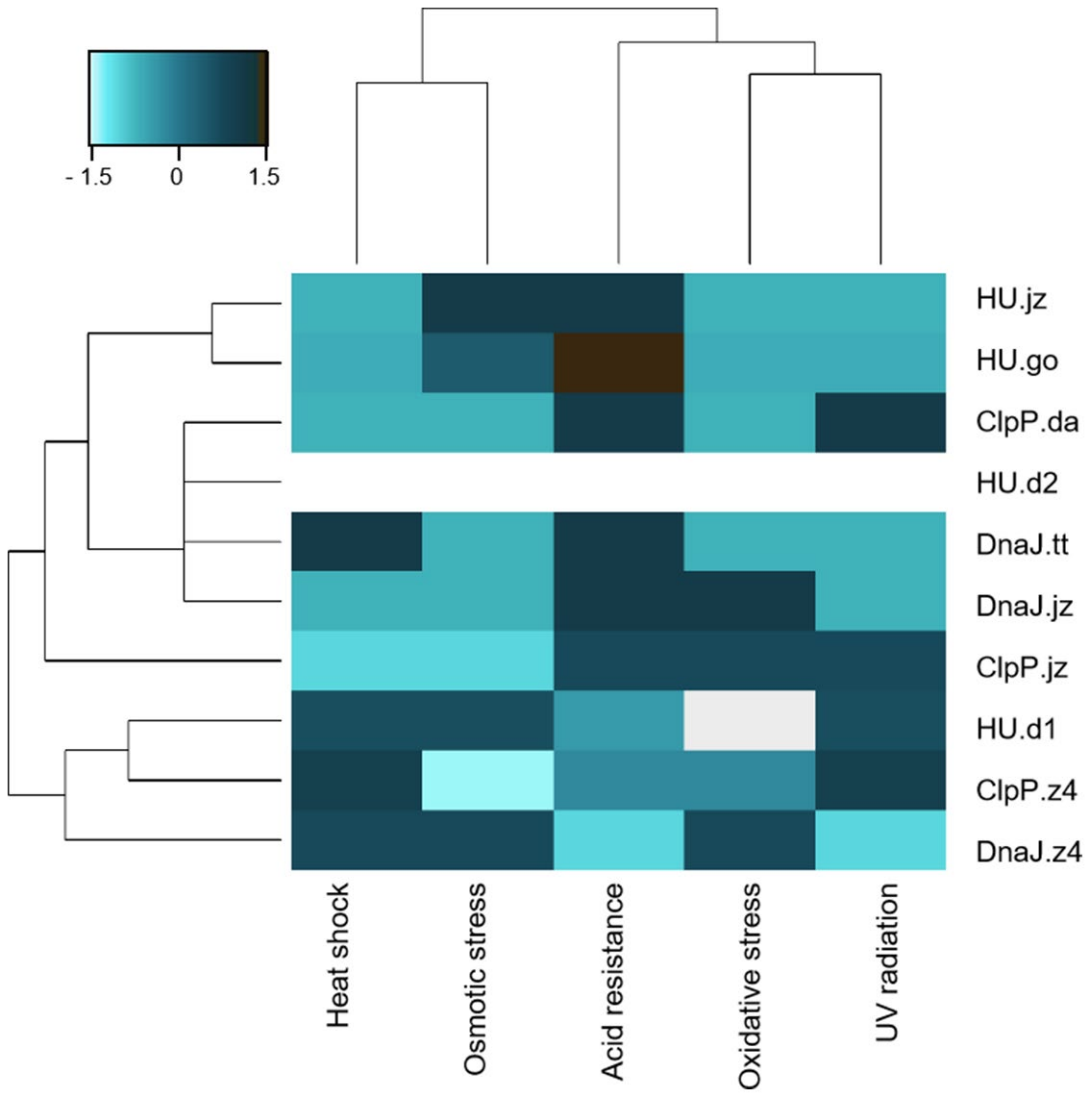
Clustering of *E. coli* clones considering functional characterization in the five stress conditions. Hierarchical cluster of *E. coli* DH10B clones carrying the pUC19 plasmid with genes *clpP*, *hup,* and *dnaJ*. Scale bar denotes the Z-score fold change. Strong color represents an increased response level to stress factors.

In a similar way, cells expressing *clpP.jz* or *dnaJ.z4* showed a positive response when exposed to three different stress factors (acidity, oxidative stress, and UV radiation for cells expressing *clpP.jz*, and heat shock, oxidative, and osmotic stress for cells expressing *dnaJ.z4*). For example, cells expressing *clpP.jz* showed an average survival percentage of around 29% in the acidity challenge (Figure 2C) and 2% in oxidative stress (Figure 2D). These values represent a fold change of 290 and 9 times, respectively, in comparison to negative controls. Moreover, UV radiation tests also showed a significantly higher survival of cells expressing *clpP.jz* compared with cells bearing empty plasmid (20 s versus 10 s) (Figure 2E; Supplementary Figure S7). In accordance with the obtained results, there are several works in the literature describing a connection between this protease and cellular damage protection in stress conditions (Guazzaroni et al., 2013; Chen et al., 2015; de Siqueira et al., 2020). For instance, a functional metagenomic study from an extremely acidic environment identified a *clpP* gene able to confer resistance to acidity and UV radiation in *E. coli* (Guazzaroni et al., 2013). Also, when this gene was used for the construction of a combinatorial acid stress-tolerance module improved the growth robustness of *E. coli* at low pH (de Siqueira et al., 2020). Additional examples in other microorganisms, such as *Bifidobacterium longum* and *Lactococcus lactis*, also show that ClpP is involved in the acid stress response (Frees and Ingmer, 1999; Wei et al., 2015). It is important to highlight that during oxidative stress, hydrogen peroxide diffuses easily into cells, increasing intramolecular concentrations (Sen et al., 2020). In the cytoplasm, the reactions produce hydroxyl radicals that can damage DNA and other biomolecules,—similar to the effects of protons during acid stress—, endangering the survival of the cell (Arcari et al., 2020; Sen et al., 2020; Figure 3). Hydrogen peroxide triggers the production of reactive oxygen species (ROS), which, in turn, oxidize proteins, resulting in the formation of misfolded proteins (Daly et al., 2007; Wang et al., 2009). These misfolded proteins must undergo degradation. Similarly, after UV radiation exposure, excited sensitizers can directly react with DNA directly by transferring electrons or energy to molecular oxygen, thus generating ROS, which in turn can damage the DNA molecule (Schuch and Menck, 2010).

On the other hand, clones expressing *dnaJ.z4* had an average survival of 0.87% in the heat shock test (Figure 2B) and 2.35% in oxidative stress (Figure 2D), representing more than 12 and 10 times of survival in respective negative control. Also, when the cells expressing *dnaJ.z4* were subjected to saline stress, they reached a 44% higher growth rate compared to untransformed *E. coli* cells (Figure 2F; Supplementary Figure S8). It should be noticed that DnaJ protein is part of the molecular response machinery to heat shock (Ghafoori et al., 2017), confirming the results found in our study. A recent work in which the *dnaJ* gene from *Bacillus halodurans* was overexpressed in *E. coli* showed improved cell thermotolerance, indicating that DnaJ overexpression was sufficient to increase resistance to high temperatures (Ghafoori et al., 2017). On the other hand, our study is the first one—to the best of our knowledge—to report DnaJ protein protection against oxidative cellular damage.

## 4. Conclusion

The approach presented here allowed us to identify five novel coding sequences conferring resistance to at least two stress conditions when expressed in *E. coli*. It should be noticed that all expressed proteins, except for HU.d2, demonstrated resistance to at least one of the tested stress conditions (Figures 2, 3). Notably, among all genetic constructs, cells expressing HU.d2 exhibited a significantly higher metabolic cost compared to the other clones (Figure 2A; Supplementary Table S6), which should explain its limited response to stress conditions. Also, it is worth mentioning that the response level varied among clones despite proteins belonging to the same family. This inconsistency may be attributed to the origin of metagenomic DNA from diverse microorganisms, where certain genes tend to be preferentially expressed in hosts possessing transcription/translation machinery more closely resembling the source organism (Gabor et al., 2004).

Clustering of *E. coli* clones stress-responses illustrated that probably the expression of each protein corresponds to specific molecular mechanisms of survival (Figure 3). Notably, UV radiation, acidity, and oxidative stress exhibit a similar response pattern, probably due to the fact that UV radiation and acidity indirectly induce oxidative stress by generating ROS (Schuch and Menck, 2010).

Despite considerable research on stress response available in the literature, the approach presented here has revealed novel aspects of this well-established response, including genes that potentially enhance resistance. On the other hand, from a biotechnological point of view, one of the identified novel coding sequences (*hu.d1*) retrieved from an acidic soil metagenome notably increased microbial tolerance to four different stress conditions, indicating its suitability for the construction of a synthetic circuit directed to expand broad bacterial resistance. Moreover, the assembly of genetic constructs with a combination of the best-identified gene candidates from our study (e.g., *hu.d1*, *clpP.jz*, and *dnaJ.z4*) should lead to improved bacterial endurance. Taken together, this work provides novel biological parts for the engineering of microbial hosts which could be used for extreme industrial applications. Still, further research is necessary to comprehend the mechanisms of action when these proteins are heterologously expressed and to understand the functional behavior of the identified genes in other hosts than *E. coli*.

## Data availability statement

The original contributions presented in the study are included in the article/Supplementary material, further inquiries can be directed to the corresponding author.

## Author contributions

JH-J: Formal analysis, Investigation, Methodology, Project administration, Writing – original draft, Writing – review & editing. EN-S: Formal analysis, Investigation, Methodology, Writing – review & editing. NS: Formal analysis, Methodology, Software, Writing – review & editing. RS-R: Conceptualization, Formal analysis, Supervision, Writing – review & editing. M-EG: Conceptualization, Funding acquisition, Supervision, Writing – original draft, Writing – review & editing.

## Footnotes

^1^ https://www.mg-rast.org/

^2^ https://blast.ncbi.nlm.nih.gov

^3^ http://www.pymol.org/

^4^ https://www.ebi.ac.uk/Tools/st/emboss_backtranseq/

^5^ http://parts.igem.org
