# Supplementary material for: Identification and functional analysis of novel protein-encoding sequences related to stress-resistance
Source: Front Microbiol. 2023 Sep 28;14:1268315. doi: 10.3389/fmicb.2023.1268315 (PMC10568318; doi:10.3389/fmicb.2023.1268315)
Supplement: Supplementary file 1 [file Data_Sheet_1.pdf]

Identification and functional analysis of novel protein-encoding sequences related to stress-resistance

Joshelin Huanca-Juarez[a,b], Edson Alexandre Nascimento-Silva[a,b,] Ninna Hirata Silva[a], Rafael Silva-Rocha[c], María-Eugenia Guazzaroni[b*]

[a]Department of Cell and Molecular Biology, Ribeirão Preto School of Medicine (FMRP) - University of São Paulo (USP) - Ribeirão Preto, SP, Brazil

[b]Department of Biology, Faculty of Philosophy, Sciences and Letters of Ribeirão Preto (FFCLRP) - University of São Paulo (USP) - Ribeirão Preto, SP, Brazil.

[c]ByMyCell Inova Simples. Av. Dra. Nadir Aguiar, 1805 – Supera Parque, Ribeirão Preto, SP, Brazil

[*]Corresponding author

Supplementary material

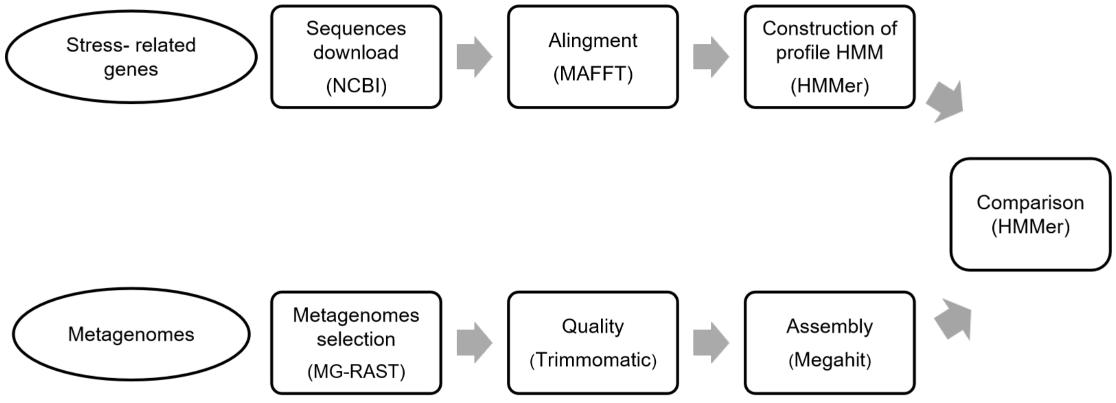

Figure S1. Overview of the pipeline for analysis and identification of potential stress-related genes.

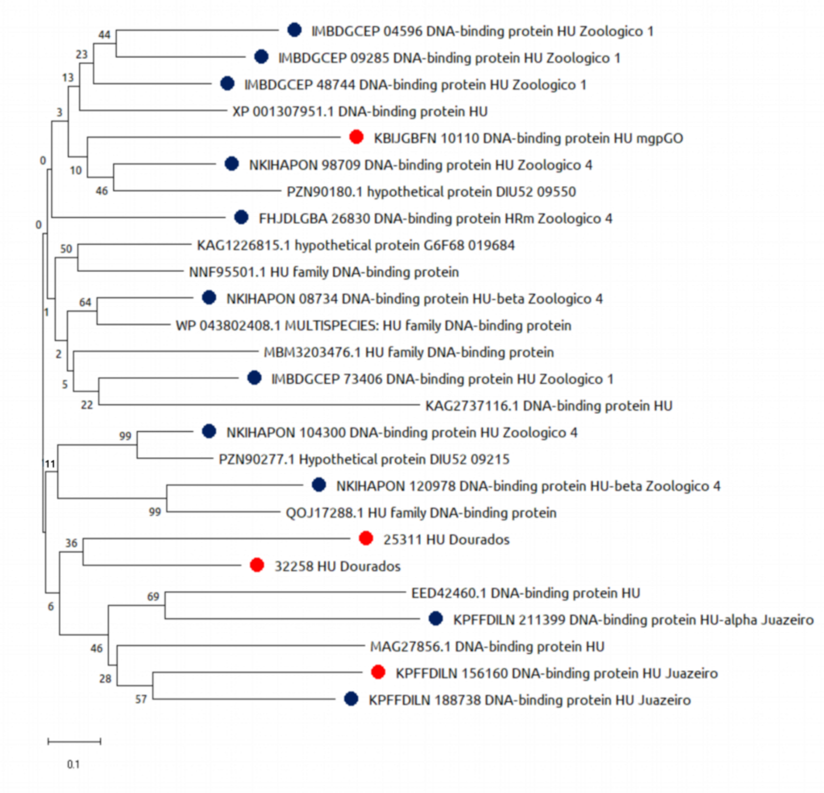

Figure S2. Dendrogram of the HU protein sequences. Dendrogram of 26 amino acid sequences of HU using the Neighbor-Joining (NJ) method. Amino acid sequences were retrieved from GenBank as indicated by accession numbers as follows: XP_001307951.1 (*Trichomonas vaginalis* G3), MBM3203476.1 (*Candidatus Woesearchaeota archaeon*), KAG2737116.1 (*Suillus brevipes* Sb2), PZN90180.1 (bacteria), QOJ17288.1 (*Phycisphaeraceae bacteria*), PZN90277.1 (bacteria), WP_0438.1 (*Arenimonas*), NNF95501.1 (*Halobacteria archaeon*), KAG1226815.1 (*Rhizopus microsporus*), EED42460.1 (*Enterocytozoon bieneusi* H348), MAG27856. 1 (*Candidatus Pacearchaeota archaeon*). The numbers on the nodes correspond to the bootstrap percentage values for 1000 replicates. The circles represent the pre-selected sequences, and the color if they were selected (red) or not (blue).

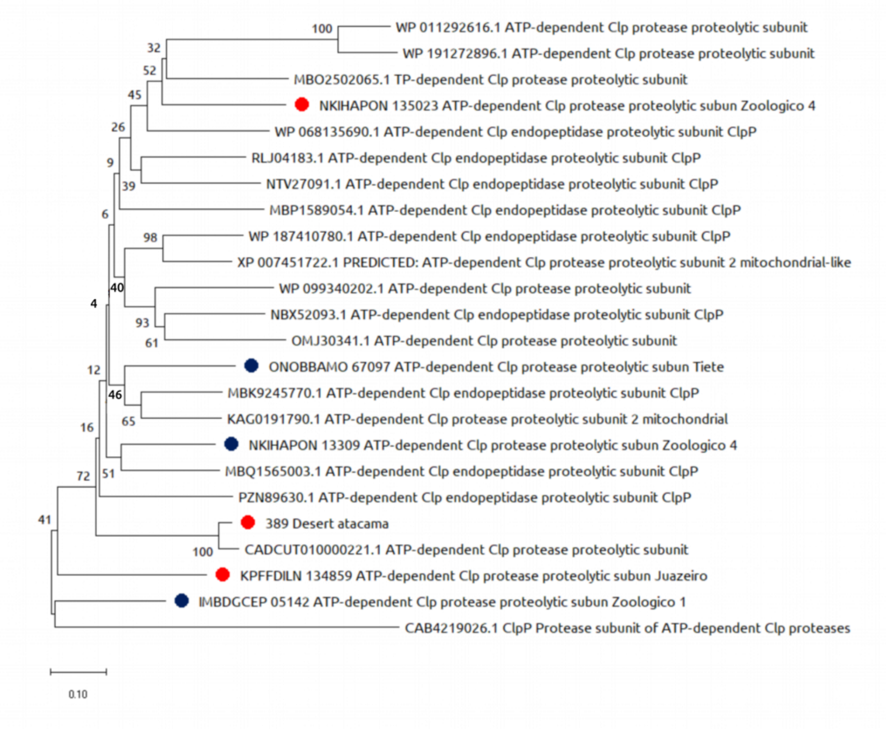

Figure S3. Dendrogram of the ClpP protein sequences. Dendrogram of 24 amino acid sequences of ClpP using the Neighbor-Joining (NJ) method. Amino acid sequences were retrieved from GenBank as indicated by accession numbers as follows: WP_011292616.1 (*Thermobifida fusca*), WP_191272896.1 (*Nocardiopsis terrae*), MBO2502065.1 (*Thermoanaerobacterales bacterium*), WP_068135690.1 (*Limnochorda pilosa*), RLJ04183 .1 (*Candidatus Aenigmarchaeota archaeon*), NTV27091.1 (*Methanothrix* sp.), MBP1589054.1 (*Kiritimatiellae bacterium*), WP_187410780.1 (*Saccharophagus* sp. K07), XP_007451722.1 (*Lipotes vexillifer*), WP_099340202.1 (*Candidautus Fonsibacter*), NBX52093.1 (*Proteobacteria bacterium*), OMJ30341.1 (*Smittium culicis*), MBK9245770.1 (*Burkholderiales bacterium*), KAG0191790.1 (*Apophysomyces* sp. BC1034), MBQ1565003.1 (*Clostridia bacterium*), PZN89630.1 (bacterium), CADCUT010000221.1 (uncultured *Rubrobacteraceae bacterium*), CAB4219026.1 (uncultured *Caudovirales* phage). The numbers on the nodes correspond to the bootstrap percentage values for 1000 replicates. The circles represent the pre-selected sequences, and the color, if they were selected (red) or not (blue).

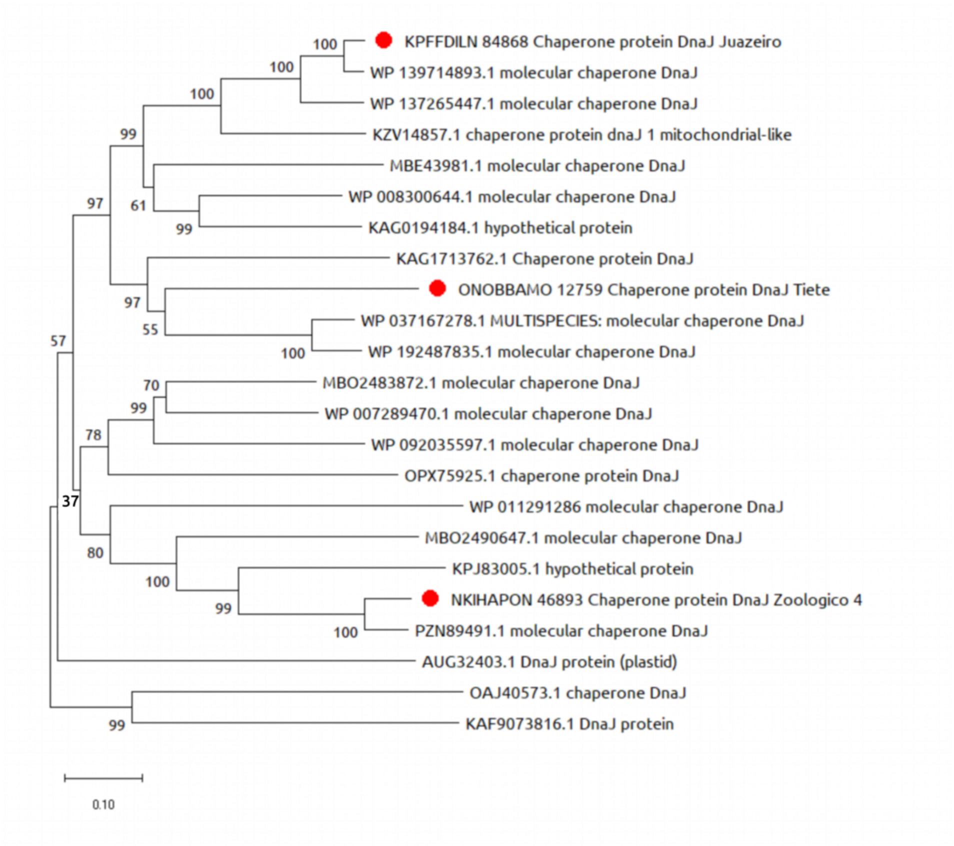

Figure S4. Dendrogram of the DnaJ protein sequences. Dendrogram of 23 amino acid sequences of the DnaJ protein using the Neighbor-Joining (NJ) method. Amino acid sequences were retrieved from GenBank as indicated by accession numbers as follows: WP_139714893.1 (*Thermomonas* sp. SY21), WP_137265447.1 (*Luteimonas gilva*), KZV14857.1 (*Dorcoceras hygrometricum*), MBE43981.1 (*Thaumarchaeota archaeon*) , WP_008300644.1 (*Candidatus Nitrosopumilus salaria*), KAG0194184.1 (*Apophysomyces* sp. BC1034), KAG1713762.1 (*Nymphon striatum*), WP_037167278.1 (unclassified *Rhizobium*), WP_192487835.1 (*Agrobacterium* sp. AGB081), MBO481 *Firmicutes bacterium*), WP_007289470.1 (*Thermosinus carboxydivorans*), WP_092035597.1 (*Planifilum fulgidum*), OPX75925.1 (*Methanosaeta* sp. PtaB.Bin018), WP_011291286 (*Thermobifida fusca*), MBO2490647.1 (*Rhodo Kthermus*J5P8.1) *Gemmatimonas* sp.SG8_23), PZN89491.1 (bacterium), AUG32403.1 (*Paulinella longichromatophora*), OAJ40573.1 (*Batrachochytrium dendrobatidis* JEL423), KAF9073816.1 (*Rhodocollybia butyracea*). The numbers on the nodes correspond to the bootstrap percentage values for 1000 replicates. The circles represent the pre-selected sequences, and the color, if they were selected (red) or not (blue).

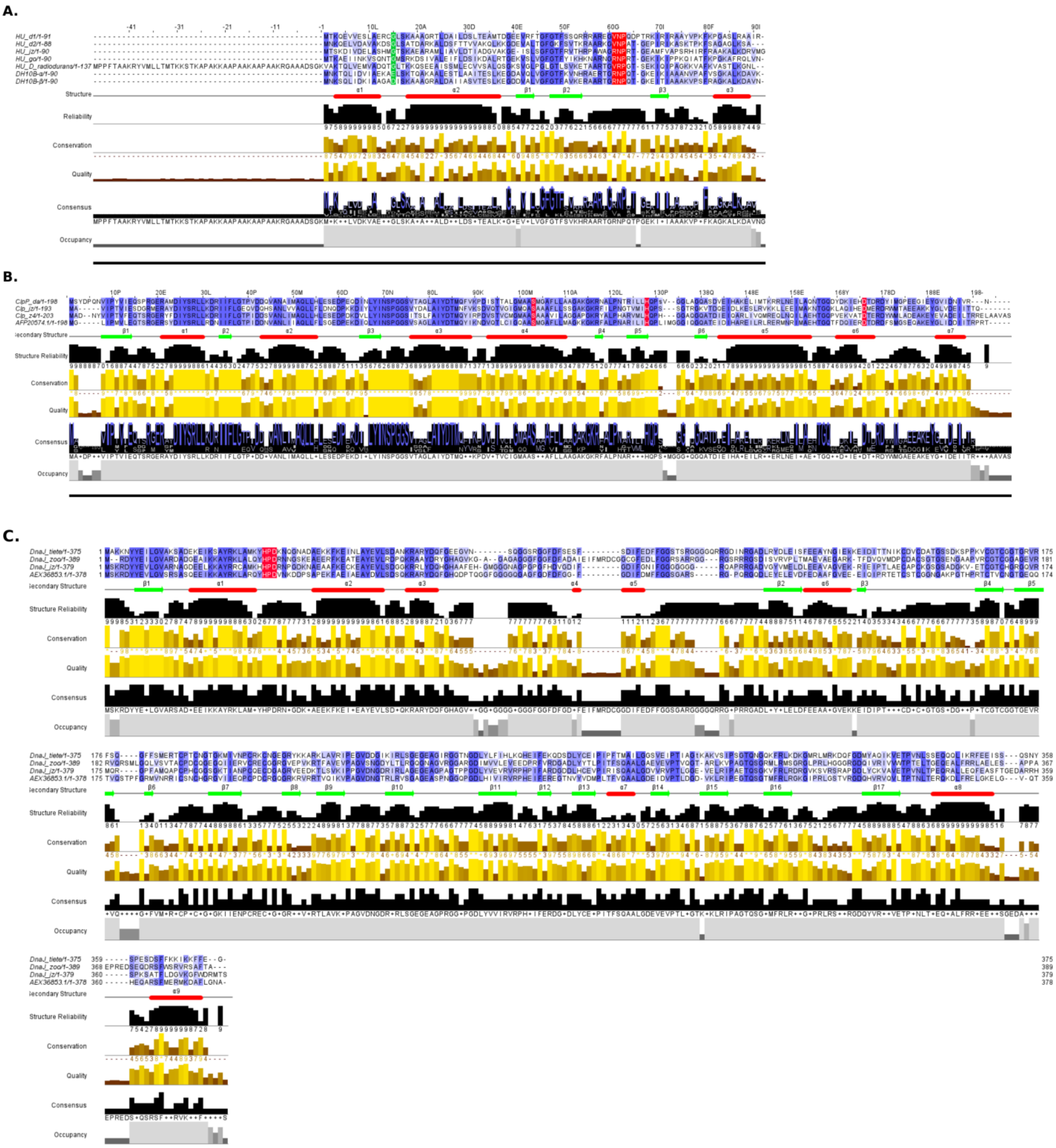

Figure S5. Multiple sequence alignment of HU, ClpP and DnaJ amino acid sequences. (A) Alignment of HU protein sequences, including the 4 selected sequences (HU.d1, HU.d2, HU.jz and HU.go), HU alpha and beta from *E. coli* DH10B and HU from *Deinococcus radiodurans*. (B) Alignment of ClpP protein sequences, including the 3 selected sequences (ClpP.da, ClpP.jz, and ClpP.z4) and ClpP of metagenomic origin (GenBank: JX219770). (C) Alignment of DnaJ protein sequences, including the 3 selected sequences (DnaJ.tt, DnaJ.z4, and DnaJ.jz) and DnaJ from *Alicyclobacillus acidoterrestris*. Residues from the catalytic site of each protein are indicated in red. Secondary structures and reliability of predicted secondary structure are shown below the sequence.

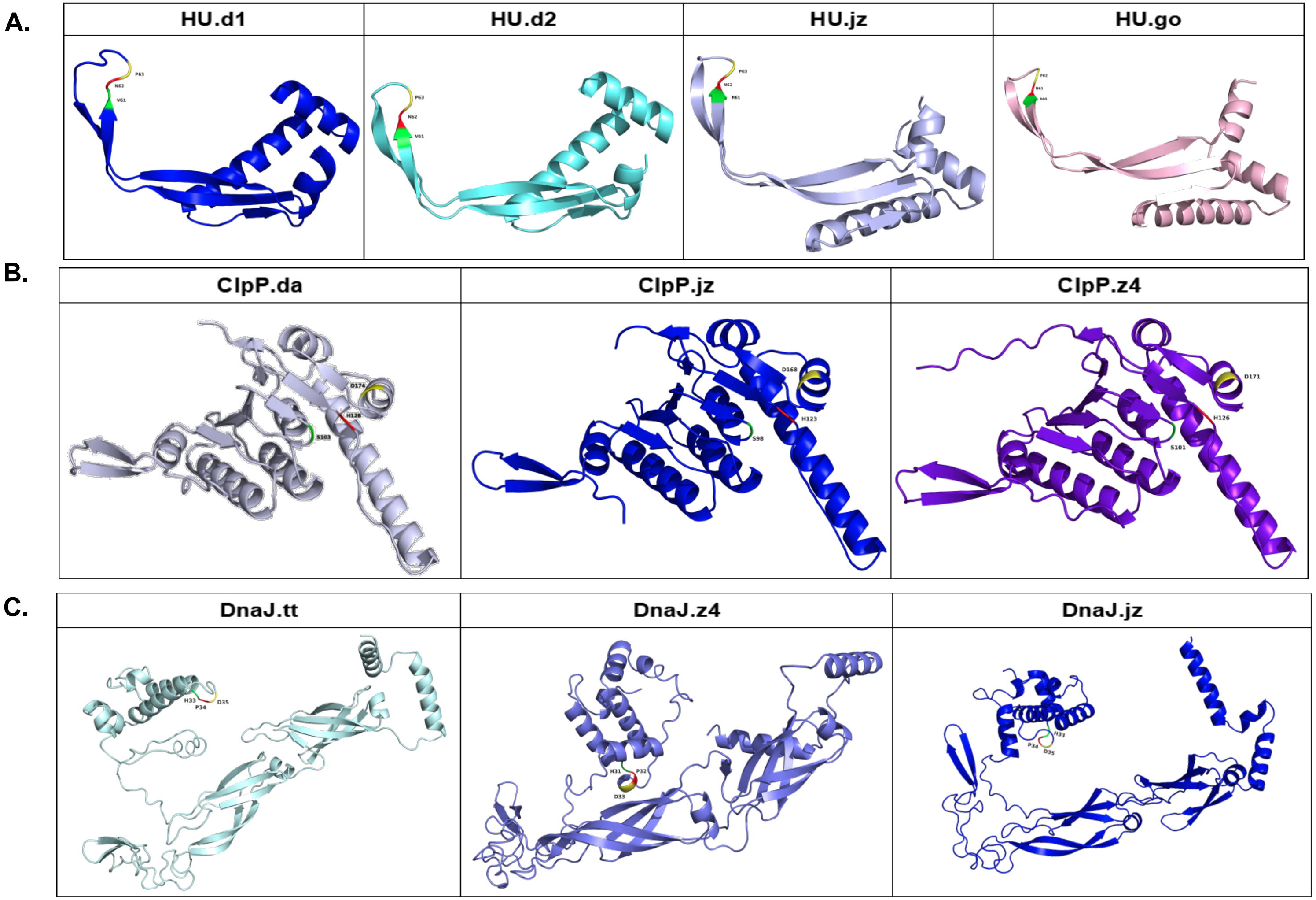

Figure S6. 3D structural model of selected proteins. The figure shows models for (A) HU, (B) ClpP, and (C) DnaJ proteins. Catalytic residues are highlighted for each protein.

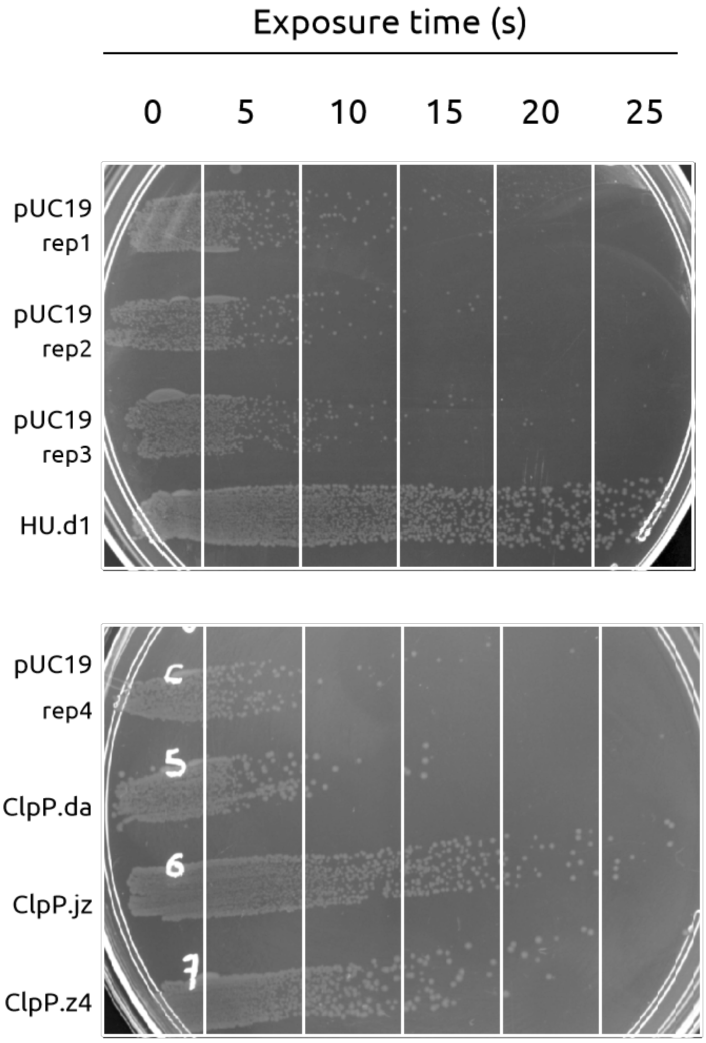

Figure S7. Effect of the retrieved genes in UV radiation resistance assays. Overnight cultures of *E. coli* DH10B, linearly spread on M9 plates, carrying the pUC19 plasmid with subcloned genes *clpP*, *hu* and *dnaJ* were irradiated with a germicidal lamp during 0, 5, 10, 15, 20, and 25 seconds. *E. coli* DH10B carrying empty pUC19 was used as a negative control. Each assay was performed at least three times using independent cultures.

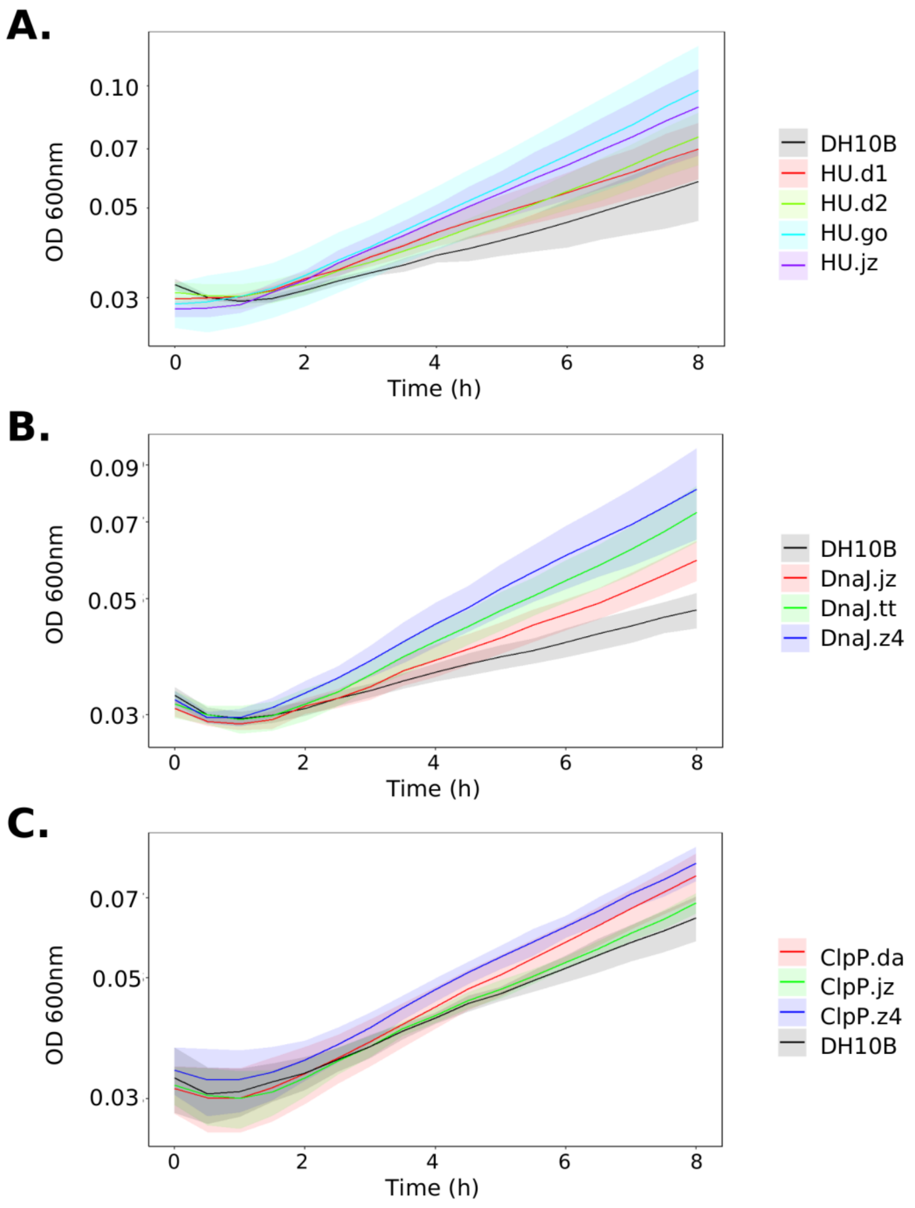

Figure S8. Survival growth curves of clones harboring plasmids containing (A) *hu*, (B) *dnaJ* and (C) *clpP* genes at 3.5% NaCl supplemented M9 medium. All graphs represent the average from four biological replicates. Standard deviation from experiments is represented as shaded regions.

Table S1. Description of metagenomes selected through the MG-RAST platform.

ID	File name	Study name	Size (MB)	Material	Condition	Location
mgp8766	AmazPluma	AmazPluma	1300	River water	Salinity	Amazon, Brazil
mgp81659	juazeiro	Microbial community urban environment with heavy metal industrial waste	299.3	Margem do rio	High heavy metals concentration levels (Zn and Cu)	Ceara, Brazil
mgp18106	mgpGO	Evaluation of microbiota in serpentine soils for nickel biomining	1400	Bulk soil	High heavy metals concentration levels (Ni)	Goias, Brazil
mgp86353	Tiete	Plas_Tiete	10400	Water	Contaminated river	São Paulo, Brazil
mgp5435	Zoologico 1	Study of composting at the São Paulo Zoo (1)	368.7	Compost	Temperature 66-67º C	São Paulo, Brazil
mgp5435	Zoologico 2	Study of composting at the São Paulo Zoo (2)	302.6	Compost	Temperature 66-67º C	São Paulo, Brazil
mgp5435	Zoologico 3	Study of composting at the São Paulo Zoo (3)	1500	Compost	Temperature 66-67º C	São Paulo, Brazil
mgp5435	Zoologico 4	Study of composting at the São Paulo Zoo (4)	1900	Compost	Temperature 66-67º C	São Paulo, Brazil
mgp11367	Desert_atacama	Atacama metagenomes	1.2	Rock	High temperature	Chile
mgp18410	Dourados	Metagenomas solos de Dourados	46.7	Soil	Acidity	Mato Grosso, Brazil

Table S2. Assembly statistics of metagenomic datasets by MegaHit software.

Study place	Time (h:m:s)	File size	Total length (bp)	N50 (bp)	Total contigs	Min (bp)	Max (bp)	Mean (bp)
Amazon	1:05:05	166.4 M	153839154	509	300170	200	105781	512
Juazeiro	0:36:37	305.5 M	277741274	399	664071	201	10585	418
Barro Alto	01:09:49	40.1 M	36771520	484	81125	200	3313	453
Tietê	02:12:23	190.9 M	178760935	635	290434	211	117691	615
Zoo 1	00:15:05	106.8 M	98014883	426	212382	200	21393	461
Zoo 2	0:12:57	94.6 M	86253073	404	201129	200	8025	428
Zoo 3	00:38:03	122.8 M	113058559	432	234957	200	57509	481
Zoo 4	1:10:48	272.4 M	250706729	452	519263	200	37665	482

Table S3. Summary of the annotation results made by the Prokka software.

Study place	Annotations	CDS	Hypothetical proteins	Non hypothetical proteins
Amazon	238677	237951	228208	9743
Juazeiro	214382	211222	165074	46148
Barro Alto	46982	46289	41059	5230
Tietê	155070	152095	113505	38590
Zoo 1	77408	75542	60420	15122
Zoo 2	65510	63935	49206	14729
Zoo 3	91203	89077	65406	23671
Zoo 4	203237	199648	142173	57475

Table S4. Summary of number of hits of different proteins found in metagenomes.

Protein	M1	M2	M3	M4	M5	M6	M7	M8	M9	M10
**DPS**	4	0	3	32	2	5	5	33	*	4
**HU**	18	98	15	84	36	38	43	93	*	15
**RBP**	1	28	4	29	7	6	6	22	*	5
**DnaA**	60	62	6	85	30	28	45	97	1	23
**GyrA**	31	35	9	64	33	20	25	74	1	29
**RecA**	186	22	5	86	24	12	26	54	*	24
**ClpA**	99	84	27	206	64	54	106	186	1	111
**ClpC**	84	78	25	183	63	42	94	177	1	103
**ClpE**	60	62	18	161	62	37	79	152	1	67
**ClpL**	50	45	19	135	42	28	62	114	1	58
**ClpP**	68	54	11	101	28	26	43	87	1	17
**ClpX**	76	64	18	167	47	34	72	143	*	71
**Cas1**	2	5	0	0	1	5	7	12	*	*
**Cas2**	0	1	0	0	0	5	7	11	*	*
**Cas9**	5	3	1	8	1	3	2	6	*	*
**DnaJ**	22	90	9	114	42	23	33	105	*	*
**DnaK**	36	45	3	81	20	27	45	58	*	*

The columns represent the following environments: (M1) Amazon River (M2) Juazeiro (M3) Serpentine soil (M4) Tietê River (M5) Zoo 1 (M6) Zoo 2 (M7) Zoo 3 (M8) Zoo 4 (M9) Desert from Atacama (M10) Dourados.

* Data not analyzed.

Table S5. Amino acid sequences of the 10 proteins selected for experimental validation.

Sequence name	Aminoacid sequence
HU_d1	MTKQEVVESLAERCGLSKAAAGRTLDAILDSLTEAMTDGEEVRFTGFGTF SSQRRRAREGVNPQDpTRKIRIRAAYVPKFKPGASLRAAIR
HU_d2	MNKQELVDAVAKDSGLSATDARKALDSFTTVVAKQLKKGDEVALTGFG KFSVTKRAARKGVNPATGEPIRIKASKTPKFSAGAGLKSA
HU_jz	MTSKDIVDELASHMGTSKAEARAMLIAVLDTIADGVAKGEISLSGFGTFRV THRPAWAGRNPRTGEAMFVAPSRHIRFRAAKALKDRVMG
HU_go	MTKAEIINKVSQNTGMSRKDSIVALEIFLDSIKDALRTGEKVSLVGFGTFYI KHKNARNGRNPRTGEKIKIPPKQIATFKPGKAFRQLVN
ClpP_da	MSYDPQNVIPYVIEQSPRGERAMDIYSRLLKDRIIFLGTPVDDQVANAIMA QLLHLESEDPEQDINLYINSPGGSVTAGLAIYDTMQFVKPDISTTALGMAA SMGAFLLAAGAKGKRNALPNTRILLHQPsVGGLAGQASDVEIHAKELIMT KRRLNEILAQNTGQDYDKIEHDTDRDYIMGPEEGIEYGVIDNIVRN
ClpP_jz	MAVIPTVIESDGRMERAYDIYSRLLKDRIIFLGEQVDQHSANLVVAQLLFL DNQDPKKDIYLYINSPGGSVYDALAIYDTMNFVKSDVQTVGIGMQASAAA FLLSSGAKGKRFILPNGTVMIHQPSSGTRGKVTDQEIDLKESLRVKKLLEEI MAKNTGQKLAQIHEDMERDRWMTAEEAKKYGLVDEIITTQ
ClpP_z4	MADNYVIPTVFEQTSRGERYFDIYSRLLKDRIIFLGTPIDDSVANLIMAQLL HLESEDPDKDVLLYINSPGGSITSLFAIYDTMQYIRPDVSTVCMGMAASAA AVILAGGAPGKRYALPHARVMLHQPHGGAQGQATDIEIQARLIVQMREQL NQILAEHTGQPVEKVATDTERDYWMLADEAKEYEVADEILTRRELAAVA S

DnaJ_tt	MAKKNYYEILGVAKSADEKEIKSAYRKLAMKYHPDKNQGNADAEKKFK EINLAYEVLSDANKRARYDQFGEEGVNSQGGSRGGFDFSESFSDIFEDFFG GSTsrgGGGQRRGDINRGADLRYDLEISFEEAYNGIEkKEIDITTNIKCDVCD ATGSSDKSPPKVCGTCGGTGRVRFSQGFFSMERTCPTCNGTGKMIVNPCR KCNGEGRYKKARKLAVRIPEGVDDGIKIRLSGEGEAGIRGGTNGDLYLFIH LKQHEIFEKQDSDLYCEIPIPFTMAILGGSVEIPTIAGtKAKVSIPSGTQNGQK FRLKDKGMRLMRKDQFGDMYAQIKVETPVNLSSEQQQLIKRFEEISSQSN YSPESDSFFKKIKKFFEG
DnaJ_z4	MRDYYEILGVARDADGEAIKKAYRKLALQYHPDRNNGSKEAEERFKEAT EAYEVLRDPQKRAAYDRYGHAGVKGAGAGAGGGFGGFDFADaleIFmrdcg GCGFEDLFGGRSARRggrgSRRRGSDISVRVPLTMAEVAEGARKTFDVQVM DPCDACSGTGSENGAAPVRCGTCGGAGEVRRVQrsmlGQLVSVTACPDCQ GEGQIIERVCRECGGRGVEPVKRTFAVEVPAGVSNGDYLTLRGQGNAGVR GGARGDIMVVLEVEEDPRFVRDGADLYYTLPITFSQAALGAEVEVPTVQG TARLKVPAGTQSGRMLRMSGRGLPRLHGGGRGDQIVRIVVWTPTELTGE QEALFRRLAELESAPPAEprEDSEQDRSFWSRVRSAFTA
DnaJ_jz	MSKRDYYEVLGVARNAGDEELKKAYRRCAMKHHPDRNPGDKNAEAAF KECKEAYEVLSDGGKRRLYDQHGHAAFEHGMGGGNAGPGpgfHDVGDIF GDIFGNIFGGGggGGRQAPRRGADVGYVMELDLEEAVAGVEKRIEIPTLAE CAPCKGSGSADGKVETCGTCHGRGQVRMQRGPFAMQAPCPHCGGSGKTI ANPCQECDGAGRVEEDKTLSVKIPPGVDNGDRIRLAGEGEAGPAGTPPGD LYVEVRVRPHPIFARDGDDLHCEVPIRISQAALGDVVRVPTLGGEVELRIP AETQSGKVFRLRDRGVKSVRSRAPGDLYCKVAVETPVNLTPEQRALLEQF EASFTGEDARRHSPKSATFLDGVKGFWDRMTS

Table S6. Growth rate and fitness cost of clones grown in M9GlycAA medium supplemented with Ap when strains contained plasmids.

Clone	Growth rate (h^{-1})	Fitness cost (%)
DH10B (reference)	0.38	0.0
HU.go	0.42	-10.5
HU.jz	0.40	-5.3
ClpP.da	0.38	0.0
ClpP.z4	0.36	5.3
DnaJ.tt	0.36	5.3
HU.d1	0.34	10.5
DnaJ.z4	0.34	10.5
ClpP.jz	0.34	10.5
HU.d2	0.28	26.3
DnaJ.jz	0.26	31.6
pUC19	0.22	42.1

Table S7. Summary of the results obtained for each stress assay.

Clone	Heat Shock (%)	Acidity (%)	Oxidative stress (%)	UV radiation (s)	Salinity (h^{-1})
**Negative control**	1.2 ± 0.10	0.1 ± 0.08	0.24 ± 0.23	8.75 ± 3.1	0.09 ± 0.02
**HU.d1**	1.20 ± 0.10	0.84% ± 0.12	0.35 ± 0.09	25	0.12 ± 0.01
**HU.d2**	0.01 ± 0.02	0.21 ± 0.21	0.18 ± 0.12	11.7 ± 2.9	0.10 ±0.03
**HU.jz**	0	9.60 ± 5.17	0.72 ± 0.49	11.7 ± 2.9	0.16 ± 0.02
**HU.go**	0.01 ± 0.02	15.48 ± 4.72	0.3 ± 0.18	11.7 ± 2.9	0.15 ±0.05
**ClpP.da**	0.3 ± 0.18	1.78 ± 0.38	0.76 ± 0.88	15 ± 10	0.13 ±0.03
**ClpP.jz**	0.64 ± 0.37	29.48 ± 4.19	2.05 ± 0.72	21.7 ± 2.9	0.12 ±0.04
**ClpP.z4**	1.08 ± 0.28	11.59 ± 7.20	3.04 ± 3.63	21.7 ± 2.9	0.12 ±0.03
**DnaJ.tt**	0.87 ± 0.30	4.42 ± 2.04	0.56 ± 0.33	21.7 ± 2.9	0.11 ± 0.02
**DnaJ.z4**	1.20 ± 0.10	0.95 ± 0.98	2.35 ± 0.16	11.7 ± 2.9	0.13 ± 0.02
**DnaJ.jz**	0.02 ± 0.04	6.97 ± 4.91	2.9 ± 0.71	11.7 ± 2.9	0.09 ±0.02
